# Diosgenin and Monohydroxy Spirostanol from *Prunus amygdalus var amara* Seeds as Potential Suppressors of EGFR and HER2 Tyrosine Kinases: A Computational Approach

**DOI:** 10.3390/ph16050704

**Published:** 2023-05-06

**Authors:** Mohammed Helmy Faris Shalayel, Ghassab M. Al-Mazaideh, Abdulkareem A. Alanezi, Afaf F. Almuqati, Meshal Alotaibi

**Affiliations:** 1 Department of Pharmacy Practice, College of Pharmacy, University of Hafr Al Batin, Hafr Al Batin 31991, Saudi Arabia; 2 Department of Pharmaceutical Chemistry, College of Pharmacy, University of Hafr Al Batin, Hafr Al Batin 31991, Saudi Arabia; 3 Department of Pharmaceutics, College of Pharmacy, University of Hafr Al Batin, Hafr Al Batin 31991, Saudi Arabia

**Keywords:** bitter almond, EGFR, HER2, molecular docking, ADEMT, molecular dynamic, MM-PBSA

## Abstract

Cancer continues to be leading cause of death globally, with nearly 7 million deaths per year. Despite significant progress in cancer research and treatment, there remain several challenges to overcome, including drug resistance, the presence of cancer stem cells, and high interstitial fluid pressure in tumors. To tackle these challenges, targeted therapy, specifically targeting HER2 (Human Epidermal Growth Factor Receptor 2) as well as EGFR (Epidermal Growth Factor Receptor), is considered a promising approach in cancer treatment. In recent years, phytocompounds have gained recognition as a potential source of chemopreventive and chemotherapeutic agents in tumor cancer treatment. Phytocompounds are compounds derived from medicinal plants that have the potential to treat and prevent cancer. This study aimed to investigate phytocompounds from *Prunus amygdalus var amara* seeds as inhibitors against EGFR and HER2 enzymes using in silico methods. In this study, fourteen phytocompounds were isolated from *Prunus amygdalus var amara* seeds and subjected to molecular docking studies to determine their ability to bind to EGFR and HER2 enzymes. The results showed that diosgenin and monohydroxy spirostanol exhibited binding energies comparable to those of the reference drugs, tak-285, and lapatinib. Furthermore, the drug-likeness and ADMET predictions, performed using the admetSAR 2.0 web-server tool, suggested that diosgenin and monohydroxy spirostanol have similar safety and ADMET properties as the reference drugs. To get deeper insight into the structural steadiness and flexibility of the complexes formed between these compounds and theEGFR and HER2 proteins, molecular dynamics simulations were performed for 100 ns. The results showed that the hit phytocompounds did not significantly affect the stability of the EGFR and HER2 proteins and were able to form stable interactions with the catalytic binding sites of the proteins. Additionally, the MM-PBSA analysis revealed that the binding free energy estimates for diosgenin and monohydroxy spirostanol is comparable to the reference drug, lapatinib. This study provides evidence that diosgenin and monohydroxy spirostanol may have the potential to act as dual suppressors of EGFR and HER2. Additional in vivo and in vitro research are needed to certify these results and assess their efficacy and safety as cancer therapy agents. The experimental data reported and these results are in agreement.

## 1. Introduction

Cancer is a leading cause of death worldwide, and traditional chemotherapy and radiation treatments can often have significant side effects [1]. This has led to a growing interest in alternative and complementary therapies for cancer [1]. In recent years, natural compounds have emerged as promising candidates for the treatment of cancer due to their ability to target specific signaling pathways involved in cancer progression [2]. There is a growing body of research exploring the use of natural compounds as alternative or complementary therapies for cancer [3].

*Prunus amygdalus var. amara* is a type of almond that contains a variety of phytocompounds that can be used to make essential oils with a potent fruity-almond aroma [4,5], as well as extracts and essential oils with potential healing properties [4,5]. Bitter almonds have been used to prevent and cure diseases for centuries [6], and recent research has associated the inclusion of almonds in the diet with elevating the levels of antioxidants, which can help protect against cancer [5]. A recent study suggested that bitter almond extract may possess anti-cancer properties by regulating the expression and immortality of MCF-7 (breast cancer cells) [4]. These extracts have been shown to exhibit anti-cancer properties, and their ability to target specific signaling pathways involved in cancer progression has received significant attention [4,6]. Despite these promising developments, it remains critical to further our knowledge of the molecular mechanisms of the action of these natural compounds as well as their efficacy in complex biological systems [6].

The anti-cancer activity of the Bitter almond active compounds demonstrated the anti-cancer impact of its active compounds in a diversity of cell lines, besides lung, breast, colon, and cervical cells [7,8,9]. The compounds induced apoptosis in a caspase-independent manner, with a dose much lower than other anti-cancer treatments [7,8,9]. In addition, these results show that the anti-cancer activity of the active compounds is dose-dependent and independent of the cell line tested, suggesting that the effect of these compounds may have a broad application to various cancer treatments [10].

Epidermal growth factor receptor (EGFR) and human epidermal growth factor receptor 2 (HER2) are two proteins that play a significant role in cancer proliferation [11,12]. EGFR is a transmembrane glycoprotein with an intracellular tyrosine kinase that serves as a receptor for extracellular protein ligands [12]. HER2 is a coreceptor that belongs to the human epidermal growth factor receptor family, which also includes EGFR, HER3, and HER4 [13]. HER2 works as an oncogene in several cancers, including breast cancer, manifesting its effect of carcinogenesis mainly by overexpression or due to gene amplification [13,14].

EGFR and HER2 are frequently overexpressed or mutated in solid tumors, such as carcinomas and gliomas [15]. EGFR drives metastasis in many ways, including paracrine loops comprising tumor and stromal cells that enable EGFR to fuel invasion across tissue barriers, survival of clusters of circulating tumor cells, and colonization of distant organs [11]. HER2 is overexpressed in 20% of breast cancer cases and is activated ligand-independently by homodimerization. In addition, HER2 is able to heterodimerize with EGFR, HER3, and HER4, which has been proposed as a mechanism of resistance to therapy for HER2 [16].

Anti-cancer drugs targeting either EGFR or HER2 have been clinically approved, but the emergence of drug resistance is nearly inevitable [11,16]. Therefore, it is important to consider the major evasion mechanisms, such as the heregulin-EGFR-HER3 autocrine signaling axis that can mediate acquired lapatinib resistance in HER2+ breast cancer models [11]. Understanding the roles of EGFR and HER2 in cancer proliferation is crucial for developing effective therapies that can target these proteins and overcome drug resistance [17,18,19]. TKIs (tyrosine kinase inhibitors) such as gefitinib, erlotinib, neratinib, and afatinib, are used to target EGFR/HER2 [17,20,21]. These four drugs have been associated with adverse impacts that can effectively influence patient health [17,20,21]. Lapatinib is a dual suppressor of EGFR and HER2 that has been used in clinical trials [19,22]. Lapatinib is an irreversible suppressor of chemotherapeutics [19,22] and was selected as a regarded drug in the current work.

The display investigation was initiated to evaluate the feasibility of new bioactive inhibitors as a means of addressing the limitations of clinically approved molecules for cancer treatment. There has been a growing interest in developing alternative cancer treatments and therapies that do not involve radiotherapy and chemotherapy, as these approaches can have negative effects on patients’ health. In response, phytocompounds are gaining recognition as promising chemotherapeutic and chemopreventive agents in cancer therapy, as they can be derived from medicinal plants and exhibit the potential to treat and prevent cancer. The present study specifically focused on the characterization and examination of the phytochemical composition of Bitter almond seed, intending to identify a popular phytocompound dual suppressor for EGFR as well as HER2 that can demonstrate anti-cancer activity via molecular docking as well as dynamic simulation investigations [2,3,7,20]. This study expands upon previous research efforts aimed at uncovering new, naturally derived bioactive compounds with anti-cancer properties, and it will be expanded to show the anti-cancer activity of the crude extract as dose-dependent on 8 cancer cell lines (phase 2).

## 2. Results and Discussion

### 2.1. GC–MS Analysis of the Extract

**Figure 1:**
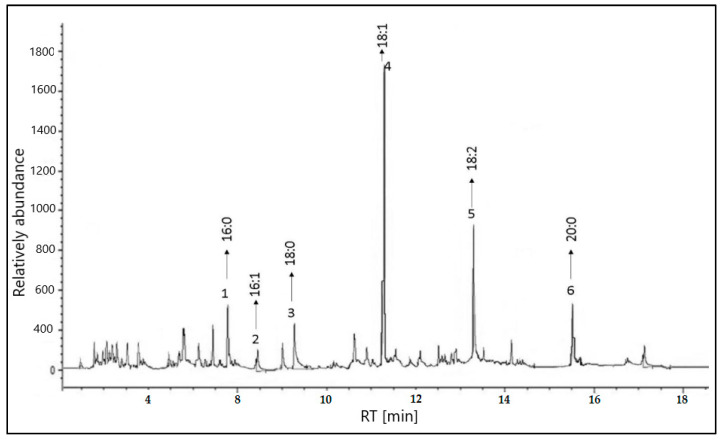
GC-MS chromatogram of *Prunus amygdalus var. amara* seeds.

The percentages of each component were determined using the Gas Chromatography (GC) technique. All identified peaks with area% greater than 0.812 were displayed, and unwanted peaks were integrated based on the standard material that was injected first. The relative percentage of each component was calculated and tabulated in Table 1 and illustrated in Figure 1. 

The GC chromatogram revealed that the presence of the following unsaturated fatty acids predominates and represents (88.60%) while the saturated fatty acids represent only 11.39%: saturated fatty acids palmitic, and stearic, along withthe unsaturated fatty acids oleic, linoleic, arachidic, and palmitoleic. It is obvious that oleic acid was the major unsaturated fatty acid (52.04%) and oleic acid was the major saturated fatty acid (6.55%). The main essential fatty acids (linoleic, and arachidic) play an important role in the development of a baby’s brain and prevent cancer [23].

### 2.2. HPLC Analysis of the Extract

**Figure 2:**
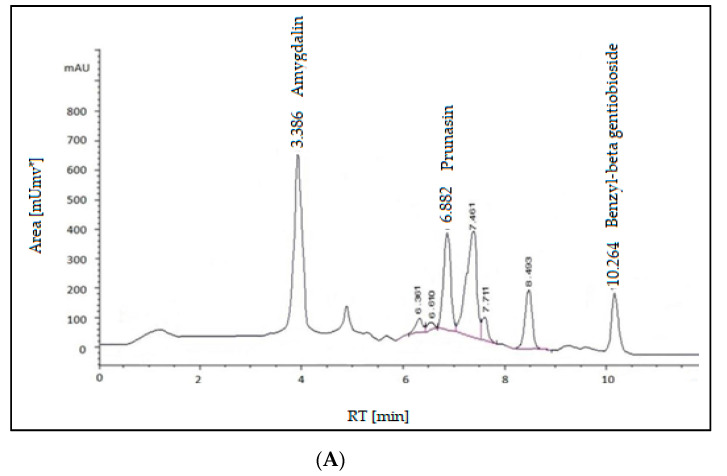

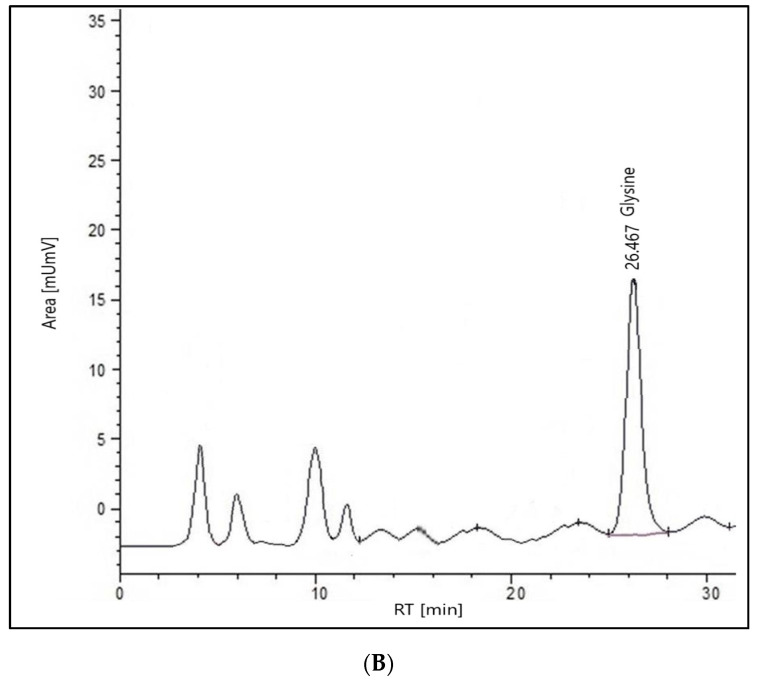
(**A**) HPLC-(UV-VIS) chromatogram of *Prunus amygdalus var. amara* seeds. (**B**) HPLC-(UV-VIS) chromatogram of *Prunus amygdalus var. amara* seeds.

The main idea of HPLC separation is based on the variation of the analyte (sample) between the mobile phase (eluent) as well as the stationary phase (fill material from the column). Depends primarily on the chemical composition of the analyte and the molecules lag while traversing the stationary phase. Identifies the specific interactions between sample molecules and packing materials at their respective times in the column. Hence, the different components of the sample are plotted at different times. Thus, the components of the sample are separated. We identify the indication unit of the analyst after finishing the column, and then the signals are transformed and recorded by a data administration system (computer program) and then displayed on a chromatogram (Figure 2A).

#### 2.2.1. Glycine 

All the standard and sample solutions were injected three times, andthe average of the three readings was taken. The main active constituent, glycine, was appearing at a retention time of 26.467 min (Figure 2B). Chromatographic analysis showed glycine with a value of 0.285 mg/mL. Glycine helps the body make glutathione, which is an important antioxidant that protects the body from cell damage, resulting in cancer prevention [24].

#### 2.2.2. Glycosides

The result of the determination of glycosides using HPLC showed the presence of the glycosides: amygdalin, prunasin, and benzyl-beta gentiobioside. All of these components appeared as follows for retention time: 3.386, 6.882, and 10.624 min, respectively. Amygdalin had the highest percentage (24.768 mg/mL), followed by prunasin (8.783 mg/mL) while benzyl-beta gentiobioside had the lowest (5.959 mg/mL). Two minor compounds, prunasin, and benzyl-beta gentiobioside, were identified along with the primary glycoside, amygdalin, and they were characterized as mandelic acid glycosides. The anti-tumor encouraging activity of these compounds was examined in both in vitro as well as in vivo assays. All the components had significant inhibition, and the Epstein-Barr virus early antigen stimulation was induced by the tumor promoter [25,26].

#### 2.2.3. Flavonoids

All the standard and sample solutions were injected three times, and the average of the three readings was taken. The major component Kaempferol showed a retention time of 26.483 min, and the minor component centaureidin appeared at a retention time of 32.447 min, allowing kaempferol and centaureidin to calculate their percentages in the formulation as 0.9903 mg/g and 0.551 mg/g, respectively. Kaempferol, a well-characterized phytonatural flavonol, is found in 80% of plant-based food products, together with broccoli, tomatoes, grapes, beans, apples, strawberries, and some plant seeds or fruits. Thus, the importance appeared as anti-metastasis, anti-angiogenesis, and anti-proliferative [27].

#### 2.2.4. Steroids

The method proceeded using the reported conditions mentioned according to the method used. All the standard and sample solutions were injected three times, and the average of the three readings was taken. Diosgenin was measured using peak area vs. concentration (mg/mL). The concentration of diosgenin was 3.087 mg/mL. Cleary showed a retention time of 8.866 min.

The steroidal diosgenin obtained from *Prunus amygdalus var. amara* seeds has been studied to execute anti-cancer activities in various human cancer cell lines [28].

### 2.3. LC-MS/MS Descriptions

The analysis of the value of energy had good flavonoids, phenolic acids, and their glycoside identifications, which led to a good understanding of the value of these compounds. The electrospray ionization in the +ve as well as the -ve ion mode, especially in combination with the MS/MS method, has quickly been the method of choice through sugar sequence evaluation. A positive ion mode electrospray ionization mass spectrum. The peak at *m/z* 456.6 corresponds to the [M+Na]^+^, and *m/z* 460.3 corresponds to the [M+NH_4_]^+^. The peak at *m/z* 353.3 corresponds to the diglucoside ion generated by the loss of DL-mandelonitrile. the fragments and RT perspective to identify amygdalin or its derivatives. On the other hand, the peak at *m/z* 456.6, 459.4 and 460.3 corresponds to amygdalin and its metabolite compound, prunasin. Also, positive ion mode fragments were found based on the existence of the aglycone peak at *m/z* 610.3–611.4 correlating to [aglycone+H]^+^, which suggested a saturated monohydroxy spirostanol skeleton [29]. In some cases, the aglycone peak seemed to be found at *m/z* 415, according to either [aglycone+H] or [aglycone+H-H_2_O]^+^, for the negative –ion mode The deprotonated ion was *m/z* 667.5 and 668.5 [M-H]^−^, which corresponds to a cyanogenic glucoside known as thebenzyl-beta gentiobioside compound.

### 2.4. Molecular Docking Studies

In this investigation, molecular docking was utilized to predict the in silico molecular interactions between phytocompounds 1–14 and their targets, namely the epidermal growth factor receptor (EGFR) as well as the human epidermal growth factor receptor 2 (HER2). The binding free energy (ΔG) of each compound was determined and compared against reference controls comprising the co-crystallized ligand tak-285 and the commercially available drug lapatinib, both of which are known inhibitors of EGFR and HER2. The results of this analysis are described in Table 2. Our goal of this analysis is to identify hit phytocompounds with free binding energies substantially similar or lower than the reference controls, as they may have the potential to serve as effective and selective inhibitors of EGFR and HER2. 

First, the AutoDock 4.2 software was used to re-dock the tak-285 co-crystallized ligand against the EGFR (3POZ.PDB) and HER2 (3RCD.PDB) proteins in order to validate the docking process parameters for the subsequent docking of 14 isolated phytocompounds. The RMSD of tak-285 was found to be 1.39 Å for EGFR (Figure S1) and 1.40 Å for HER2 (Figure S2), values that are within the acceptable range for using docking to predict the binding affinity of small molecules. These findings may demonstrate the reliability of the docking process parameters and support the use of docking as a tool for predicting the binding affinity of isolated phytocompounds against the EGFR and HER2 proteins. 

Fourteen phytocompounds were compared to reference structures against EGFR and HER2. Diosgenin and monohydroxy spirostanol from this group showed EGFR binding energies of −9.77 kcal/mol as well as −9.74 kcal/mol, respectively, which were comparable to those of the reference drugs tak-285 and lapatinib. (Table 2). These values were similar to those of the reference drugs tak-285 (−9.71 kcal/mol) and lapatinib (−9.64 kcal/mol). For the HER2 receptor, diosgenin and monohydroxy spirostanol revealed the lowest binding energy values, −9.19 kcal/mol and −10.10 kcal/mol, respectively, as well as among the 14 phytocompounds (Table 2). These values were similar to those of the reference drugs tak−285 (−9.21 kcal/mol) and lapatinib (−8.90 kcal/mol). Among these compounds, diosgenin exhibited a slightly lower binding energy value than the reference-bound ligands for the HER2 receptor. These findings suggest that diosgenin and monohydroxy spirostanol may have the potential to be effective phytocompounds (inhibitors) of both EGFR and HER2 and warrant further investigation.

#### Interaction Examines the Hit Phytocompounds

**Figure 3:**
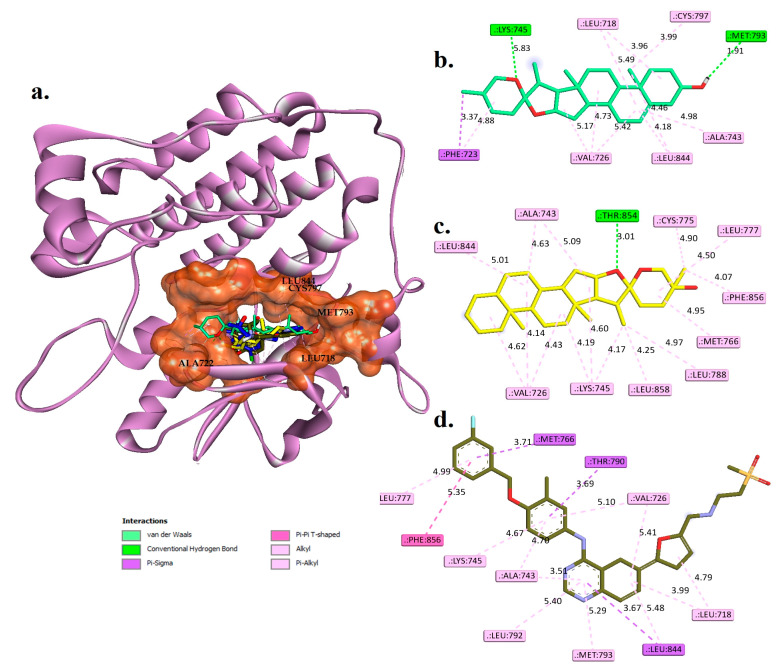
(**a**) The docked structures of diosgenin (coloured green C and red O), monohydroxy spirostanol (coloured yellow C and red O), lapatinib (coloured army green C, red O, stune N, orange S, and cyan F), and the original co-crystallized tak-285 (coloured dark blue C, red O, stune N, green CL, and cyan F) are superimposed into the active binding site (coloured rust) of EGFR (3POZ.PDB). (**b**–**d**) Two-dimensional interaction views of the key and surrounding amino acids with diosgenin, monohydroxy spirostanol, and lapatinib, respectively.

**Figure 4:**
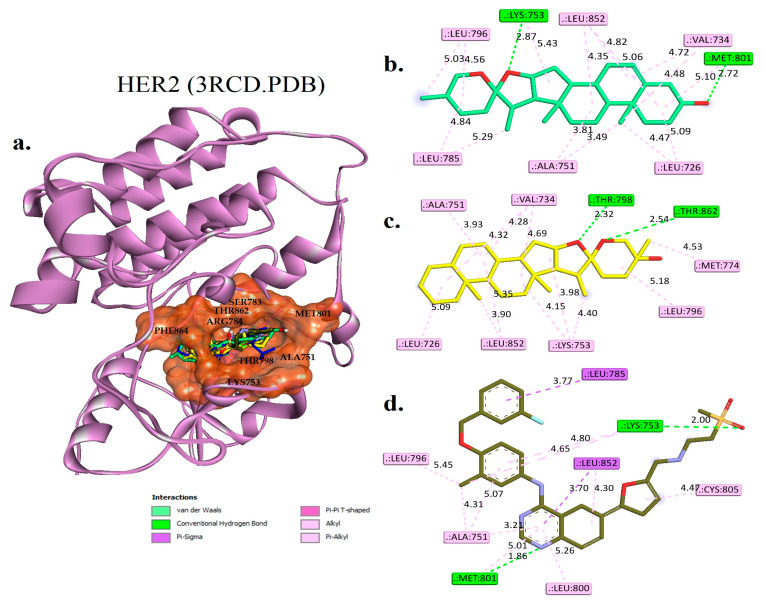
(**a**) The docked structures of diosgenin (colored green C and red O), monohydroxy spirostanol (colored yellow C and red O), lapatinib (colored army green C, red O, stune N, orange S, and cyan F), and the original co-crystallized tak-285 (colored dark blue C, red O, stune N, green CL, and cyan F) are superimposed into the active binding site (colored rust) of HER2 (3RCD.PDB). (**b**–**d**) Two-dimensional interaction views of the key and surrounding amino acids with diosgenin, monohydroxy spirostanol, and lapatinib, respectively.

The potential of the hit phytocompounds as active compounds was evaluated through analysis of non-covalent interactions with target receptors. The best-docked pose for these compounds is depicted in Figure 3 for EGFR and Figure 4 for HER2. In addition, the interactions of these compounds were compared to those of tak-285 and lapatinib, reference drugs for EGFR and HER2, to further understand their potential mechanisms of action. Table 3 and Table 4 compare the stability as well as interactions of diosgenin and monohydroxy spirostanol with amino acid residues at the active binding sites of EGFR and HER2 receptors via hydrogen bonding, Pi Sigma, and hydrophobic interactions. These findings are compared to those for the reference drug lapatinib and the original co-crystallized stability of tak-285. 

Diosgenin and monohydroxy spirostanol displayed favorable docking scores with common amino acids in the active binding site of EGFR, with binding energies of −9.77 kcal/mol and −9.74 kcal/mol, respectively (Table 2).

The molecular docking results in Figure 3 and Table 3 suggest that both diosgenin and monohydroxy spirostanol may have favorable interactions with the EGFR receptor, as evidenced by their hydrogen bond and pi-sigma bond formations, as well as their hydrophobic interactions with specific amino acid residues. These interactions may contribute to the affinity of these compounds for the EGFR receptor. 

Diosgenin forms hydrogen bonds with LYS745 and MET793 at distances of 1.81 Å and 1.91 Å, respectively, and also forms a pi-sigma bond with PHE723. In addition, diosgenin has hydrophobic interactions with LEU718, VAL726, PHE723, CYS799, LEU844, and ALA743. Monohydroxy spirostanol forms a hydrogen bond with THR854 at a distance of 2.01 Å and has hydrophobic interactions with VAL726, ALA743, LYS745, MET766, CYS775, LEU777, LEU788, LEU844, PHE856, and LEU858. These interactions may contribute to the affinity of these compounds for the EGFR receptor. In comparison, the reference drugs tak-285 and lapatinib have less favorable interactions with the EGFR receptor.

Tak-285 forms hydrogen bonds with LEU777, THR790, and MET793 at distances of 3.45 Å, 3.60 Å, and 2.78 Å, respectively, and also forms pi-sigma bonds with LEU718 and LEU844. It has hydrophobic interactions with VAL726, ALA743, LYS745, PHE766, CYS775, LEU788, LEU792, PHE856, and LEU858. Some of these amino acid residues, such as LYS745, MET793, and LEU844, also interact with diosgenin and monohydroxy spirostanol. However, the interactions of tak-285 with these residues may be less favorable than those of the phytocompounds, as the distances of the hydrogen bonds are longer and tak-285 does not form any pi-sigma bonds.

Lapatinib does not form any hydrogen bonds, but it does have hydrophobic interactions with MET766, THR790, and LEU844. Some of these amino acid residues, such as MET793 and LEU844, also interact with diosgenin and monohydroxy spirostanol (see Figure 3 and Table 3). However, lapatinib does not form any pi-sigma bonds or hydrogen bonds, which may make its interactions with these residues less favorable than those of the phytocompounds.

A molecular docking analysis was conducted to compare the binding interactions of diosgenin, monohydroxy spirostanol, tak-285, and lapatinib with the HER2 protein. The results of the analysis, presented in Figure 4 and Table 4, provide insight into the molecular interactions among these compounds and the HER2 protein at the active binding site.

Diosgenin had significant hydrogen bond interactions with LYS753 and MET801, with distances of 2.87 Å and 2.72 Å, respectively. These interactions may be significant because they involve amino acid residues that have been identified as key binding sites in the HER2 protein. In addition, diosgenin exhibited hydrophobic interactions with LEU726, VAL734, ALA751, LEU785, LEU796, and LEU852. These hydrophobic interactions may also participate in the binding affinity of diosgenin for the HER2 protein.

Similarly, monohydroxy spirostanol had hydrogen bond interactions with THR798 and THR862, with distances of 2.32 Å and 2.54 Å, respectively. These interactions may be significant because they involve amino acid residues that have been previously identified as important for binding to the HER2 protein. Monohydroxy spirostanol also had hydrophobic interactions with LEU726, VAL734, ALA751, LYS753, MET774, LEU796, and LEU852. These hydrophobic interactions may contribute to the highbinding affinity of monohydroxy spirostanol for the HER2 protein.

The original co-crystallized pose of tak-285 had hydrogen bond interactions with THR798, MET801, and THR862, with distances of 3.17 Å, 2.69 Å, and 2.77 Å, respectively. These interactions involve amino acid residues that have been identified as important for binding to the HER2 protein. Tak-285 also had pi-sigma interactions with LEU800 and LEU862 and hydrophobic interactions with VAL734, ALA751, LYS753, LEU785, LEU796, and PHE864. These interactions may participate in the overall binding affinity of tak-285 for the HER2 protein.

The reference drug lapatinib had hydrogen bond interactions with LYS753 and MET801, with distances of 2.00 Å and 1.86 Å, respectively. These interactions involve amino acid residues that have been identified as key binding sites in the HER2 protein. Lapatinib also had pi-sigma interactions with LEU785 and LEU852, and hydrophobic interactions with ALA751, LYS753, LEU796, LEU800, CYS805, and LEU852. These interactions may contribute to the binding affinity of lapatinib for the HER2 protein.

The molecular docking analysis carried out in this study suggests that diosgenin and monohydroxy spirostanol may have a similar affinity for binding and molecular interactions with EGFR and HER2 proteins as the reference medicines tak-285 and lapatinib. However, to fully evaluate the potential of these compounds as therapeutic agents, it is necessary to consider not only their binding properties but also their pharmacological properties and safety. In silico ADMET analysis can provide valuable insights into the pharmacological profiles of diosgenin and monohydroxy spirostanol and help predict their potential as hit candidates. In addition, conducting molecular dynamics studies can give a more detailed understanding of the binding mechanisms of these ligands. Together, these in silico analyses can provide a comprehensive evaluation of the suitability of diosgenin and monohydroxy spirostanol as therapeutic agents.

### 2.5. ADMET Prediction

In Table 5, we present the ADMET and drug-likeness predictions of four compounds: diosgenin, monohydroxy spirostanol, TAK-285, and lapatinib. The predictions were made using the admetSAR 2.0 web-server tool, which employs several different algorithms to analyze various properties of the compounds [24,30].

As shown in the first column, diosgenin has an Fsp3 score of 0.926 and monohydroxy spirostanol has an Fsp3 score of 1, both indicating good hydrophobic properties similar to TAK-285 (Fsp3 score of 0.269) and lapatinib (Fsp3 score of 0.172), all compounds passing theLipinski Rule of five, which is considered a basic requirement for drug-like compounds. Furthermore, none of the compounds was flagged as PAINS (pan-assay interference compounds) or BMS (Bristol-Myers Squibb) rule violators, indicating that they have not been associated with poor pharmacological properties.

Regarding the absorption, our ligands diosgenin and monohydroxy spirostanol have similar poor Caco-2 Permeability (−4.805, −4.805 respectively) compared to TAK-285 (Caco-2 Permeability of −5.505) and lapatinib (Caco-2 Permeability of −5.707), and their Pgp-inhibitor and HIA predictions were relatively better than lapatinib (Pgp-inhibitor: 0.281, 0.993, 0.992, 0.999, HIA: 0.005, 0.002, 0.009, 0.006, respectively). Notably, TAK-285 is more likely to be a substrate for P-glycoprotein efflux pumps (Pgp-substrate of 0.973), which may lower its absorption efficiency. However, it has a higher BBB penetration (0.912) than the other three compounds, indicating a potential for brain-targeting drugs.

The predicted clearances (CL) for diosgenin, monohydroxy spirostanol, TAK-285, and lapatinib were 23.332, 22.718, 8.791, and 3.997 (unit: L/h/kg), respectively. The predicted half-life (T1/2) for diosgenin, monohydroxy spirostanol, TAK-285, and lapatinib were 0.023, 0.08, 0.59, and 0.066 (unit: h), respectively. These predictions indicate that diosgenin, monohydroxy spirostanol, and TAK-285 have a relatively longer half-life and faster clearance compared to lapatinib.

Regarding toxicity, the AMES toxicity, rat oral acute toxicity, and carcinogenicity predictions for diosgenin, monohydroxy spirostanol, TAK-285, and lapatinib were 0.053, 0.019, 0.632, and 0.536, 0.748, 0.67, 0.637, and 0.388, 0.188, 0.274, 0.108, and 0.022 (unitless), respectively. These values indicate that diosgenin and monohydroxy spirostanol have lower toxicity predictions compared to TAK-285 and lapatinib, indicating a lower potential for toxicity.

In general, the hit phytocompounds diosgenin and monohydroxy spirostanol may have good drug-like properties according to ADMET and drug-likeness predictions. They have good hydrophobic properties, passed the Lipinski rule, have no identified PAINS or BMS rule violators, have relatively better Pgp-inhibitor and HIA predictions, and have lower toxicity predictions compared to TAK-285 and lapatinib. However, further analysis, such as molecular dynamics simulations, is needed to fully understand the behavior and potential of these compounds as drugs. It is also important to note that the predictions made using the ADMET Predictor 2.0 web server are only a tool to aid in the discovery and development process and should not be the sole basis for decision-making. Further in vitro and in vivo studies are necessary to fully evaluate the safety and efficacy of these compounds as potential therapeutics.

### 2.6. Molecular Dynamic Simulations

To further validate the association of diosgenin and monohydroxy spirostanol with the catalytic binding sites of EGFR and HER2, the stability of the docked complexes was investigated and compared to that of tak-285 and lapatinib. To accomplish this, 100 ns MD simulations were carried out by GROMACS 2016 for the four ligand complexes with their corresponding proteins. RMSD and RMSF of the protein, as well as ligand backbone atoms, were computed for each system to dictate their structural stability.

The average RMSD of the EGFR and HER2 complexes with tak-285, lapatinib, diosgenin, and monohydroxy spirostanol provides important insights into the structural stability and potential of these complexes as hit candidates. 

**Figure 5:**
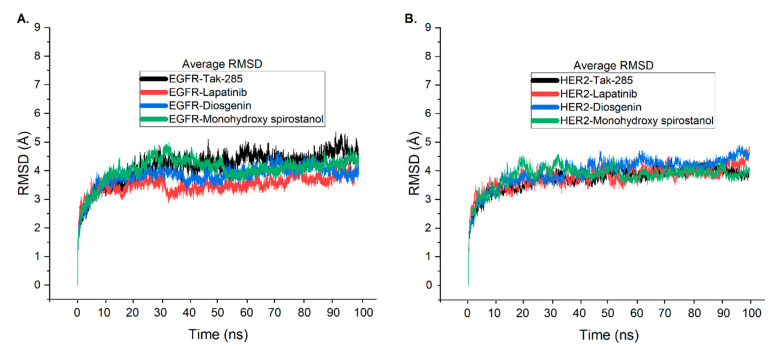
Comparison of the average RMSD values of EGFR and HER2 complexes with tak-285 (black), lapatinib (raspberry), diosgenin (sapphire), and monohydroxy spirostanol (fern). (**A**) RMSD values of EGFR complexes. (**B**) RMSD values of HER2 complexes.

As seen in Figure 5A, the EGFR-tak-285 complex reaches a stable state after 30 ns with an RMSD value of 4.2 Å and fluctuates within the range of 3.5–5.2 Å until the end of the simulation. In contrast, the EGFR-lapatinib complex starts the simulation with a lower RMSD value than the EGFR-tak-285 complex and reaches stability after 32 ns with an RMSD value of 3.1 Å, continuing to fluctuate within a narrow range of 2.7–4 Å until the simulation comes to an end.

The EGFR-diosgenin complex also reaches stability after 32 ns with a slightly lower RMSD value of 3.8 Å, slightly higher than the EGFR-lapatinib complex, and continues to fluctuate within a narrow range of 2.9–4.6 Å until the end of the simulation. 

In contrast, the EGFR-monohydroxy spirostanol system takes slightly longer to reach the stable state, around 37 ns, with an average RMSD value of 4.3 Å. Notably, the system continued to fluctuate within a lower range than the EGFR-tak-285 system, with RMSD values ranging from 3.4 to 4.6 Å until the end of the simulation.

These results provide evidence for the robust binding of diosgenin and monohydroxy spirostanol to the active binding site of EGFR. The lower RMSD values of these complexes compared to the co-crystallized ligand tak-285 and the reference drug lapatinib suggest that they are more structurally stable and may have a higher potential as drug candidates. Additionally, the fact that the RMSD values of diosgenin and monohydroxy spirostanol fall within the same range as those of lapatinib suggests that they may have similar potential as hit candidates. It is important to note that the stability of the protein-ligand complex has a direct impact on the binding affinity and efficacy of a drug candidate, as a more stable complex is less likely to dissociate from the protein and therefore more likely to effectively inhibit its activity. Therefore, the results of this study offer valuable insights into the structural stability of these complexes, which can be further used to make predictions about the binding affinity and efficacy of these compounds as EGFR inhibitors. 

In Figure 5B, it’s clear that the HER2-tak-285 complex reaches a stable state after 33 ns with an average RMSD value of 3.7 Å as well as fluctuates within a range of 3.3–4.6 Å until the simulation is over. On the other hand, the HER2-lapatinib complex reaches stability after 33 ns with an average RMSD value of 3.6 Å and continues to fluctuate within a relatively narrow range of 3.1–4.6 Å throughout the simulation.

When comparing the structural stability of the HER2-diosgenin and HER2-monohydroxy spirostanol complexes to the co-crystallized ligand tak-285 and the reference drug lapatinib, it is observed that the HER2-diosgenin complex reaches stability after 47 ns with a lower RMSD value of 3.9 Å. However, the system continues to fluctuate within a relatively wide range of 3.8–4.8 Å until the end of the simulation. Similarly, the HER2-monohydroxy spirostanol complex reaches stability at around 37 ns with an average RMSD value of 3.8 Å and continues to fluctuate within a range of 3.3–4.4 Å throughout the simulation.

Overall, the findings show that although all the complexes are stable, the HER2-diosgenin and HER2-monohydroxy spirostanol complexes have slightly higher RMSD values than the co-crystallized ligand tak-285 and the reference compound lapatinib. Furthermore, the narrow RMSD range observed in the HER2-lapatinib complex suggests that it may exhibit a higher potential as a drug candidate when compared to the other complexes. Overall, the results of this study gave valuable insights into the average structural stability of these complexes and their potential as drug candidates against EGFR and HER2, and they highlights the importance of using MD simulation as a tool for evaluating the predictability of protein-ligand complexes and in the prediction of hit candidate molecules. This can be useful for identifying potential hit candidates among a pool of compounds and for optimizing the design of novel or new drugs. Furthermore, by comparing the simulation results to the experimentally determined binding data, it can be used as a tool for validating the results of computational studies. This can help to increase the reliability of the predictions made by computational methods and, thus, the identification of potential drug candidates that would be more likely to succeed in the clinic.

The utilization of RMSF analysis in MD simulations is a valuable tool for evaluating the structural stability and flexibility of protein-ligand complexes [30]. In this study, RMSF analysis was employed to further investigate the residual flexibility of the EGFR and HER2 structures after complex formation with tak-285, lapatinib, diosgenin, and monohydroxy spirostanol to gain important insights into the structural stability and potential of these complexes as hit candidates.

**Figure 6:**
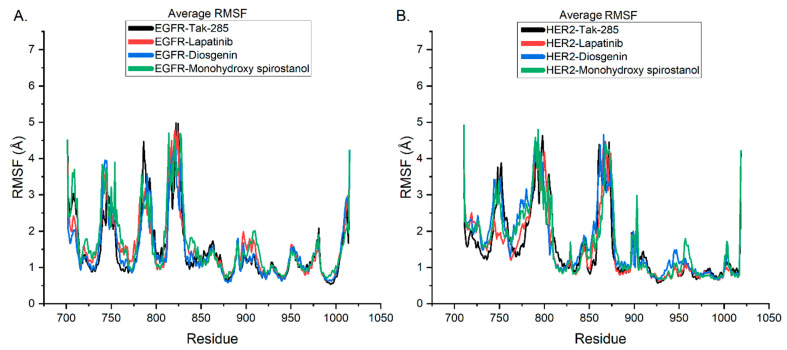
Comparison of the average RMSF values of EGFR and HER2 complexes with tak-285 (black), lapatinib (raspberry), diosgenin (sapphire), and monohydroxy spirostanol (fern). (**A**) RMSF values of EGFR complexes. (**B**) RMSF values of HER2 complexes.

The average RMSF values of each residue’s oscillation during the 100 ns MD simulation were computed and presented in Figure 6. The average RMSF plot in Figure 6A illustrates minimal fluctuation in certain loop places of the protein structure for the EGFR complex. It was observed that the EGFR protein structure after complexation with diosgenin and monohydroxy spirostanol displayed low flexibility, comparable to that of the co-crystallized ligand tak-285 and reference ligand lapatinib. This suggests that the interacting phytocompounds did not significantly affect the stability of the EGFR protein.

In Figure 6B, it was noticed that the HER2 protein structure after complexation with diosgenin and monohydroxy spirostanol displayed slightly higher flexibility compared to that of the co-crystallized ligand tak-285 and the reference ligand lapatinib. However, the difference in flexibility is not significant and does not affect the stability of the HER2 protein.

Further, the study highlighted that the loop regions of the EGFR and HER2 proteins, which are known to take part in protein-ligand interactions, were relatively stable after complexation with diosgenin and monohydroxy spirostanol. This suggests that these compounds can form stable correlations with the key residues in the active site of the proteins. This is crucial for their potential as hit candidates. Furthermore, the findings of the RMSF examination were consistent with the findings of the RMSD analysis. This provides further evidence for the robust binding of diosgenin and monohydroxy spirostanol to the active binding sites of both EGFR and HER2.

It is worth noting that the RMSF values of lapatinib also fall in the same range as those of diosgenin and monohydroxy spirostanol for both EGFR and HER2. This suggests that lapatinib, diosgenin, and monohydroxy spirostanol are similarly stable in their interactions with EGFR and HER2, and thus, they may have similar potential as drug candidates.

The outcomes of this research provide valuable insights into the structural stability and flexibility of the EGFR and HER2 complexes with tak-285, lapatinib, diosgenin, and monohydroxy spirostanol. The RMSF analysis revealed that these compounds can form stable interactions with the active site of the proteins. In addition, they did not significantly affect the stability of the EGFR and HER2 proteins. These findings may be used to estimate the binding affinity and efficacy of diosgenin and monohydroxy spirostanol as dual EGFR/HER2 kinase inhibitors. They highlight the importance of using RMSF analysis as a tool in drug discovery.

The results of the MM-PBSA calculations, as presented in Table 6, provide valuable insights into the binding energies of the ligands to the active sites of the EGFR and HER2 receptors. The overall binding energies (ΔG_Binding_) for the ligands are within the range of −62.73 to −80.24 kJ/mol, indicating that all of the ligands have a strong binding affinity for the receptors. Furthermore, the energy contributions from different interactions can be analyzed to improve our understanding of the molecular mechanisms of bonding.

It is clear from Table 6 that the major contributor to the binding energy for all the systems is the van der Waals interaction, which ranges from −50.93 to −78.99 kJ/mol. This suggests that the major driving force behind the action of ligands on receptors is the physical interaction between the atoms of the ligand and the receptor. Additionally, electrostatic interactions, which range from −28.05 to −13.74 kJ/mol, also make a significant contribution to the binding energy. This highlights the importance of electrostatic bindings in the binding of ligands to receptors.

The polar and non-polar solvation energies for the ligands are relatively low, as expected, and range from −18.59 to −11.33 and −25.98 to −14.23 kJ/mol, respectively. This suggests that the solvent does not play a key role in the binding of the ligands to the receptors. The data from the MM-PBSA calculations are in agreement with the RMSD and RMSF results, which indicate that diosgenin and monohydroxy spirostanol have strong binding interactions with both EGFR as well as HER2 receptors. The binding free energy values for diosgenin and monohydroxy spirostanol are comparable to those of the co-crystallized ligand tak-285 and reference ligand lapatinib, suggesting that they have similar binding affinities to both receptors. The electrostatic energy values for diosgenin and monohydroxy spirostanol are also similar to those of tak-285 and lapatinib, indicating that the ligands have similar interactions with the electrostatic potential of the receptors. However, the van der Waals energy values for diosgenin and monohydroxy spirostanol are slightly more negative than tak-285 and lapatinib, implying that they may have slightly different interactions with the van der Waals forces of the receptors. Overall, these results suggest that diosgenin and monohydroxy spirostanol may have the potential to act as dual inhibitors of both EGFR and HER2. Further studies, such as in vitro and in vivo assays, are required to confirm these findings. In addition, these studies are utilized to further quantify the binding affinity and efficacy of these compounds as EGFR and HER2 inhibitors. This study highlights the importance of using computational methods, such as RMSD, RMSF, and MM-PBSA, in the early stages of drug discovery to predict the structural stability and potential of protein-ligand complexes.

## 3. Materials and Methods

### 3.1. Collection and Preparation of Prunus amygdalus var amara Seeds Extract

The *Prunus amygdalus var amara* seeds were obtained from Egypt. The plant has been characterized and matched with the voucher specimen (Eg-N. S42217) by the Cairo University Herbarium. 

### 3.2. Instruments and Preparation of Prunus amygdalus var amara Seed Extraction 

#### 3.2.1. GC-MS Analysis

The plant material was obtained in a 100-mL conical flask as well as mixed with absolute ethanol using a solid/liquid ratio of 1:2 (*m*/*v*) for the extraction of the phytochemicals. Reflux was used during the extraction, which took place in a water bath at 34.4 °C. Following extraction, we used a vacuum rotating evaporator to evaporate the liquid extract at 50 °C in order to remove extra ethanol. The extract that resulted was dried completely before being stored in a desiccator [31].

An Agilent 6890 gas chromatograph with mass spectrometric detector was used with a direct capillary interface as well as fused silica capillary column PAS-5ms (30 m × 0.32 mm × 0.25 μm film thickness). The oil being investigated was injected under these conditions. Helium was utilized as the carrier gas at an estimated 1.0 mL/min in pulsed split-lowmode. The solvent incubation timewas 3 min, and the injection size has to be 1.0 μL. The mass spectrometric detector has been operated in electron effect ionization mode with anionization energy of 70 e.v. scanning via *m/z* 50 to 500. The ion supply temperature was 230 multiplier voltage (EM 5 voltage) and sustained at 1250 v out of auto-tune [31].

#### 3.2.2. HPLC System-Glycine

For the purpose of obtaining of plant seed powder, one gram of the seeds in powder form was soaked in 100 mL of hexane in an evaporator for one hour. The soaked seeds have been extracted employing an ultrasound wave and 5 mL of an extracting solution (ethanol: 0.2% metaphoric acid *v*/*v*) for 1 h from 0.5 g of ground up flesh. The resulting mixture was centrifuged at 10,000 rpm over 5 min at 4 °C, as well as the supernatant was obtained. Utilizing a concentrator for 4 h, the 0.8 mL supernatant had been dried.The samples were transferred to an HPLC glass vial as well as securely sealed after being filtered through a 0.22 m nylon syringe filter. Finally, 0.5 μL of the obtained samples were then added to an HPLC [31].

The strategy of chromatography was accompanied by injection into an HPLC system along with a specific column (100 × 2.1 mm, 1.7 μm C18), thermostated at 16 °C. Data were monitored using the Chemstation (Agilent, Santa Clara, CA, USA) chromatography data processing system. The parameters of the mobile phase system have been used as follows:

**Table:** 

**Flow Rate** **0.4 mL/min**	**A**	**B**
Time	Methanol: Formic acid (10%: 0.1%)	Methanol: Formic acid (50%: 0.1%)
0–6.5	10–30	90–70
6.5–7	30–100	70–0
8–8.5	100–10	0–90
8.5–12.5	10	90

Fluorescence is detected using a detector by excitation at 335 nm and emission at 440 nm. Retention was used to distinguish amino acids [32].

#### 3.2.3. Glycosides 

The powdered seeds (0.5 g) were ground and weighed along with a conical flask (90 mL). After that, 20 mL of ethanol was added, and extractions were performed in a shaking water bath (37 °C) in terms of water extraction. Then, extracts were purified, and ethanol was evaporated from the filtrate by a rotary evaporator. Diethyl ether (5 mL) was added to the dried sample, as well as the mixture was vortexed 1 min at room temperature to precipitate the important phytonutrients (amygdalin, prunasin, and benzyl-beta gentiobioside). The ether solvent was allowed to evaporate overnight in a fume hood, as well as the precipitated compounds were dissolved in 10 mL of water and organized for HPLC analysis.

The method of chromatography was placed on an OmniSpher C18-column (250 × 4.6 mm inner diameterand 5 μm, Varian-USA). The contents of the mobile phase were described as the following:
**Flow Rate** **0.8 mL/min****A %****B %**Time0.1% phosphoric acid: waterMethanol 100% HPLC—Grade09550–0.55–2595–750.5–280–9020–102–4.510–6090–404.5–850–6050–408–140100

The rate of flow was 0.8 mL/min. The volume of injection for samples as well as standards was 20 μL. The compounds were isolated at room temperature. Among individual runs, a 10-min re-equilibration period was used. UV/vis spectra were measured with a detection wavelength of 280 nm [33].

#### 3.2.4. Flavonoids

The extraction procedure takes 5 g of the powdered seeds in a solvent, a combination mixture of 99% aqueous methanol as well as 1% hydrochloric acid, and an efficient collection of ultrasound (UAE) and microwave (MAE) assisted extraction. Moreover, 1 g of the lyophilized dehydrated powders was mixed with 4 mL of the extraction chemicals, which containing methanol combined with hydrochloric acid (99: 1 (*v*/*v*), and after that, it was placed in a microwave, at 450 W for 15 s. Thereafter, each mixture was introduced in a vial protected against light and sonicated inside an ultrasound bath at 258 °C with a steady frequency of 35 kHz for 15 min. Following sonication, the lyophilized’ solutions were left at room temperature for about 1 h, centrifuged at 3500 rpm for 20 min, and then each extract was deposited in a laboratory flask. The samples were then examined by an HPLC system (Waters 2996) along with a photodiode array detector. The reverse-phase Supelcosil™ LC-18 column that was used is 15 cm long, has a 4 mm internal diameter, and containsoctadecyl silane particles of 5 μm diameter [31,34].

#### 3.2.5. Steroids

The powdered seeds (100 mg) were extracted in 200 mL of 70% methanol for 3 h at 60 °C using a magnetic stirrer. The extracted material was lyophilized, then dissolved in (1:1) methanol as well as filtered via a 0.22-µm nylon syringe filter, ChemLand-Poland, before HPLC assessment.

The Agilent 1200 HPLC device consisted of two LC-20AD pumps, degasser DGU-26A7, a micromixer along with 0.5–2.6 mL for HPLC-ELSD, with controller CBM-20A, also thermostat (CTO-20AC), an autosampler (SIL 20ACYR), in addition to an ELSD 3300 (Alltech Associates, Deerfield, IL, USA), a nitrogen generator, and an evaporative light scattering detector. Data was collected and handled by Chemstation software, version3.b32. The mobile phase is composed of A (99.9% water and 0.1% formic acid (*v*/*v*)) and B (99.9% acetonitrile and 0.1% formic acid (*v*/*v*)). Separation was first carried out on a Discovery C-18 column,150 mm 2.1 mm, 3 µm, using gradient program I: 0 min—20% B, 27 min—33.5% B, 45 min—100% B. The column temperature has to be 20 °C, and the flow rate has to be 0.2 mL/min. The volume of the injection was 1 µL [31,35].

### 3.3. Molecular Docking

#### 3.3.1. Protein Structure Preparation

The 3D structures of the EGFR (PDB ID: 3POZ) [36] and HER2 (PDB ID: 3RCD) [37] complexes with the co-crystallized ligand tak-285 were selected as protein targets for further analysis. These structures were obtained from the Protein Data Bank (PDB) (http://www.rcsb.org, accessed on 8 January 2023) in PDB format [38]. The approved cancer treatment drug lapatinib, which is known to inhibit both EGFR and HER2 targets, was selected as a reference drug for a comparative study [39].

The 3D structures of the protein targets were preprocessed to prepare them for molecular docking studies. This process involved the elimination of heteroatoms and non-essential water molecules using the Biovia Discovery Studio Visualizer (Biovia, 2020) [40] and subsequently saving the structures in PDB format. In addition, missing amino acids in the target structures were incorporated using the YASARA web-server tool [41]. The ionization states of titratable amino acid groups were subsequently calculated at pH 7.4 using the H++ web-server tool [42]. The resulting output was then transformed into PDBQT format using the AutoDock Tools version 1.5.6 tools [43], which included adding polar hydrogen atoms and Kollman charges. The active binding site coordinates for the docking studies were identified from the potential ligand binding domain regions of the obtained crystal structures.

#### 3.3.2. Ligand Preparation

In this study, the 2D structure of the reference drug lapatinib (PubChem ID: 208908) was taken from the PubChem database (https://pubchem.ncbi.nlm.nih.gov, accessed on 8 January 2023) to perform a molecular docking study. The structure of tak-285 was extracted from its respective protein structures as deposited in PDB, and its conformational change, if any, was taken into consideration. The 14 isolated compounds were drawn using the ChemDraw JS web page (https://chemdrawdirect.perkinelmer.cloud/js/sample/index.html, accessed on 8 January 2023) and saved in the structural data file (SDF) format. To attain the most stable conformation of each ligand, energy minimization was conducted by the Universal Force Field (UFF) and a Conjugate Gradient (CG) optimization algorithm with 1000 steps. The optimization process was carried out using the open-source babel software, and the resulting structures were saved in PDB format. Subsequently, using AutoDock tools version 1.5.6, Gasteiger charges were connected to the ligands, and the ligands were uploaded in PDBQT format, ready for molecular docking simulations.

#### 3.3.3. Molecular Docking Preparation

The molecular docking analyses of the fourteen phytocompounds were conducted to evaluate their binding affinity and molecular interactions with the EGFR and HER2 proteins. The AutoDock 4.2 Release 4.2.6 program [43,44] was utilized for the docking calculations, and the compounds were docked against reference controls, including the co-crystallized ligand tak-285 and the commercially available drug lapatinib. The Lamarckian genetic algorithm was employed to optimize the binding poses of the compounds, and the ligands were set to be flexible while macromolecules were rigid. 

The identified compounds were docked into the active binding sites of the EGFR and HER2, with the grid box focused on the center of the ligand in the original EGFR and HER2 structures. The grid box size was 40 × 40 × 40 along with the X, Y, and Z axes for both enzymes, and the coordinates of the central grid point of the map have been set to be 18.74, 31.83, and 11.62 for EGFR and 12.48, 2.96, and 28.01 for HER2. The number of runs had to be set to 150, and the maximum number of evaluations was set to 25,000,000. The default values were utilized for all other parameters to ensure consistency in the calculations. The binding energies and properties of the different phytocompounds after docking were analyzed and studied to determine their potential as therapeutic agents.

### 3.4. ADMET Prediction

In this study, we used admetSAR 2.0 [45], a web-based tool for predicting ADMET (Absorption, Distribution, Metabolism, Excretion, and Toxicity) properties, to evaluate the potential of two hit phytocompounds, diosgenin and monohydroxy spirostanol, as candidates for drug development. The ADMET properties of interest were selected based on the common properties known to be important for oral bioavailability, such as oral absorption, blood-brain barrier penetration, and CYP450 metabolism. The reference compound used in the analysis was lapatinib, a commercially available drug that has been clinically used as a tyrosine kinase inhibitor.

The methodology for using admetSAR 2.0 consisted of the following steps: (1) the compounds of interest were uploaded to the web server in SMILES format, (2) the relevant ADMET properties were selected for prediction, and (3) the results were obtained in the form of predicted values along with their corresponding confidence levels. The predictions were generated using multiple algorithms and models available on admetSAR 2.0, which included multiple linear regression, decision trees, and artificial neural networks.

To validate the predictions, we compared the predicted ADMET properties of diosgenin and monohydroxy spirostanol against the reported values of lapatinib. Additionally, we also performed in silico toxicity and drug-likeness prediction using the same server. The results of these predictions were used to evaluate the potential of diosgenin and monohydroxy spirostanol as potential hit candidates and to identify potential areas for further optimization.

### 3.5. Molecular Dynamics

In this research, we aimed to assess the stability and binding behavior of selected phytocompounds in the active sites of EGFR and HER2 proteins using MD—dynamics simulations. We used the crystal structures of EGFR and HER2 complexed with tak-285 as the starting point for our simulations. The GROMACS software package (version 2016.3) and the Gromos96 54a7 force field [46] were employed to perform the MD simulations on a nanosecond time scale. The GROMOS96 54a7 force field has been shown to yield accurate results for investigating protein dynamics and ligand binding behavior, as reported in the literature [47]. This force field has been validated through simulations of the folding equilibrium of two β-peptides with distinct dominant folds using three different force fields, including GROMOS 54A7 [47].

To prepare the systems for simulation, we generated protein and ligand topology files with appropriate tools. The protein topology file was created by the GROMACS software, pdb2gmx, whereas the ligand topology file has been created by the PRODRG server (“http://davapc1.bioch.dundee.ac.uk/cgi-bin/prodrg, accessed on 15 December 2022”). The systems have been solvated by using the TIP3P (Transferable Intermolecular Potential along with Three Points) water model, as well as counterions could be added to neutralize the charge. The systems were then minimized by the steepest descent integrator to 50,000 maximum optimization process steps as well as a 0.01 energy step size. The v-rescale coupling technique was then used to equilibrate the designed to minimize processes for 100 ps at 310 K in the steady number of particles, volume, as well as temperature (NVT) outfit. A 100-ps equilibration period was then completed using the Berendsen pressure linkage method at 1.0 bar with a steady number of particles, pressure, and temperature (NPT) [48,49]. 

After the temperature and pressure equilibrations, MD simulation runs were performed for the models for 100 ns each at 1 bar and 310 K. The short-range non-bonded interactions cut-off was set at 1.2 nm, while long-range electrostatic interactions were treated using the Particle Mesh Ewald (PME) algorithm [50]. The LINCS algorithm was used to constrain the bonds with hydrogen atoms [51]. During the molecular dynamics (MD) simulations, a time step of 2 fs was utilized for all simulations. To ensure accurate and reliable results, the coordinates were reset at regular intervals of 5000 steps, which is equivalent to 10 ps, as a part of MD data processing. This approach was implemented to maintain the integrity of the simulations and obtain robust outcomes. To analyze the MD trajectories, we used various tools and techniques such as Root Mean Square Fluctuation (RMSF), Root Mean Square Deviation (RMSD), and hydrogen bond analysis to assess the strength and stability of the interactions between the phytocompounds and the proteins.

To visually represent the results, molecular graphics images were produced using Biovia Discovery Studio Visualizer (Biovia, 2020), and graphs were prepared using OriginPro 2021 (version 9.8.0.200). These images and graphs provide a detailed representation of the systems and the interactions taking place, providing a deeper understanding of the results of the simulations.

### 3.6. Binding Free Energy Calculation Using MM/PBSA

In this study, we utilized the molecular mechanics Poisson-Boltzmann surface area (MM-PBSA) method [52], a widely used approach for calculating binding free energy from snapshots of molecular dynamics (MD) trajectories. The binding free energies of hit phytocompounds and controls against EGFR and HER2 were analyzed during the equilibrium phase of the MD simulations by taking snapshots at an interval of 100 ps from 90 to 100 ns, using the g_mmpbsa tool of Gromacs [53,54].

The binding free energy of the ligand-protein complex in solvent was calculated by subtracting the sum of the total energies of the separated protein (G_protein_) and ligand (G_ligand_) in solvent from the total free energy of the protein-ligand complex (G_complex_) according to the following equation [54]:

ΔG_binding_ = G_complex_ − (G_protein_ + G_ligand_)

The free energy for each state (complex, protein, and ligand) was calculated by adding the free energy of solvation (G_solvation_) and the average molecular mechanic’s potential energy in vacuum (EMM) [54]:

G_x_ = E_MM_ + G_solvation_

The molecular mechanic’s potential energy was calculated in a vacuum as the sum of bonded interactions (E_bonded_) and non-bonded interactions (E_non-bonded_), where E_bonded_ included bond, angle, dihedral, and improper interactions and Enon-bonded included van der Waals (E_vdw_) and electrostatic (E_elec_) interactions [54]:

E_MM_ = E_bonded_ + E_non_bonded_ = E_bonded_ + (E_vdw_ + E_elec_)

The solvation free energy (G_solvation_) was estimated as the sum of electrostatic solvation-free energy (G_polar_) and a polar solvation-free energy (G_non-polar_) [54]:

G_solvation_ = G_polar_ + G_non-polar_

The electrostatic solvation free energy (G_polar_) was computed using the Poisson-Boltzmann equation [52], while the polar solvation-free energy (G_non-polar_) was estimated from the solvent-accessible surface area (SASA) using the following equation:

Gnon-polar = γSASA + b

where γ is a coefficient related to the surface tension of the solvent and b is a fitting parameter. The values for the coefficient and fitting parameter used in this study were: γ = 0.02267 Kj/mol/Å² or 0.0054 Kcal/mol/Å² and b = 3.849 Kj/mol or 0.916 Kcal/mol.

It is important to note that the ΔE_bonded_ was assumed to be zero in this calculation [55]. The results of this binding free energy calculation provide insight into the energetics of the protein-ligand interactions and can aid in the identification of potential hit candidates. These findings were experimentally validated in our previous study [56]. 

## 4. Conclusions

In conclusion, this study suggests that diosgenin and monohydroxy spirostanol may have the potential to act as dual suppressors of both EGFR and HER2. The molecular docking analysis conducted in this study reveals that these compounds may exhibit similar binding affinity and molecular interactions with EGFR and HER2 proteins as the reference drugs tak-285 and lapatinib. The binding free energy values for diosgenin and monohydroxy spirostanol were comparable to those of the co-crystallized ligand tak-285 and the reference ligand lapatinib, suggesting that they have comparable binding affinities for both enzymes. 

Diosgenin and monohydroxy spirostanol had electrostatic as well as van der Waals energy values that were similar to those of tak-285 as well as lapatinib. This suggests that the ligands interact similarly with the electrostatic potential and van der Waals forces of the receptors.. In silico ADMET and molecular dynamics studies were also used to provide valuable insights into the pharmacological profile and interaction mechanisms of these compounds. The results indicate that diosgenin and monohydroxy spirostanol have good drug-like properties and strong binding interactions with EGFR and HER2 receptors. However, it is important to note that further in vitro as well as in vivo studies are necessary to fully evaluate their safety and efficacy as potential therapeutics. This study highlights the importance of using computational methods in the early stages of drug discovery to predict the structural stability and potential of protein-ligand complexes. The results of this study provide a promising starting point for further investigation of diosgenin and monohydroxy spirostanol as dual inhibitors of EGFR and HER2. These findings are consistent with the experimental data.

## Figures and Tables

**Table 1:** GC-MS phytoconstituents analysis of major *Prunus amygdalus var. amara* seeds compounds.

Peak no.	Retention Time (TR)	Type	Area %	Component Name	Percentage Contains %
1	7.832	(C16:0)	6.550	Palmitic	6.550
2	8.488	(C16:1)	0.812	Palmitoleic	0.812
3	9.267	(C18:0)	4.843	Stearic	4.843
4	11.316	(C18:1)	52.042	Oleic	52.042
5	13.233	(C18:2)	27.512	Linoleic	27.512
6	15.512	(C20:0)	8.241	Arachidic	8.241

**Table 2:** Free binding energy (ΔG) of isolated phytocompounds **1–14** and reference controls against EGFR (3POZ) and HER2 (3RCD) targets.

#	Target	EGFR (3POZ.PDB)	HER2 (3RCD.PDB)
		Docking Score (kcal/mol)	Docking Score (kcal/mol)
1	Amygdalin	−7.57	−6.15
2	Arachidic Acid	−6.46	−5.87
3	Benzyl-beta gentiobioside	−6.8	−6.03
4	Centaureidin	−8.26	−7.07
5	Diosgenin	−9.77	−10.1
6	Glycine	−5.03	−4.27
7	Kaempferol	−8.46	−7.1
8	Linoleic Acid	−6.99	−5.73
9	Monohydroxy spirostanol	−9.74	−9.19
10	Oleic Acid	−6.45	−5.84
11	Palmitic Acid	−5.99	−5.47
12	Palmitoleic Acid	−5.92	−5.5
13	Prunasin	−8.14	−6.14
14	Stearic Acid	−6.2	−5.53
	Tak-285 (control)	−9.71	−9.21
	Lapatinib (control)	−9.64	−9.76

**Table 3:** Molecular interactions analysis between hit phytocompounds, controls, and EGFR (3POZ.PDB).

Phytocompounds	Hydrogen Bond Interactions		Pi-Sigma	Hydrophobic Interaction
	Residues	Distances (Å)		
Diosgenin	LYS745 and MET793	1.81 and 1.91	PHE723	LEU718, VAL726, PHE723, CYS799, LEU844, and ALA743
Monohydroxy spirostanol	THR854	2.01	-----	VAL726, LYS745, MET766, ALA743, LEU777, LEU844, CYS775, LEU788, PHE856, and LEU858
Tak-285 (control)	LEU777, THR790, and MET793	3.45, 3.60, and 2.78	LEU718 and LEU844	VAL726, ALA743, LYS745, PHE766, CYS775, LEU788, LEU792, PHE856, and LEU858
Lapatinib	----	----	MET766, THR790, and LEU844	LEU718, VAL726, ALA743, LYS745, LEU777, LEU792, MET793, LEU844, and PHE856

**Table 4:** Molecular interactions analysis between hit phytocompounds, controls, and EGFR (3POZ.PDB).

Phytocompounds	Hydrogen Bond Interactions		Pi-Sigma	Hydrophobic Interaction
	Residues	Distances (Å)		
Diosgenin	LYS745 and MET793	1.81 and 1.91	PHE723	LEU718, PHE723, VAL726, ALA743, CYS799, and LEU844
Monohydroxy spirostanol	THR854	2.01	-----	VAL726, ALA743, MET766, LYS745, CYS775, LEU844, LEU777, LEU788, PHE856, and LEU858
Tak-285 (control)	LEU777, THR790, and MET793	3.45, 3.60, and 2.78	LEU718 and LEU844	VAL726, ALA743, LYS745, PHE766, CYS775, LEU788, LEU792, PHE856, and LEU858
Lapatinib	----	----	MET766, THR790, and LEU844	LEU718, VAL726, ALA743, LYS745, LEU777, LEU792, MET793, LEU844, and PHE856

**Table 5:** The ADMET and drug-likeness profiles of diosgenin, monohydroxy spirostanol, tak-285, and lapatinib were predicted using the admetSAR 2.0 web-server tool.

ADMET Prediction	Compound			
	Diosgenin	Monohydroxy Spirostanol	Tak-285	Lapatinib
Fsp3	0.926	1	0.269	0.172
Lipinski Rule	Accepted	Accepted	Accepted	Accepted
PAINS	0	0	0	0
BMS Rule	0	0	0	0
Caco-2 Permeability	−4.805	−4.805	−5.505	−5.707
Pgp-inhibitor	0.281	0.993	0.992	0.999
Pgp-substrate	0.001	0.001	0.973	0.995
HIA	0.005	0.002	0.009	0.006
BBB Penetration	0.701	0.187	0.912	0.043
CL	23.332	22.718	8.791	3.997
T1/2	0.023	0.08	0.59	0.066
AMES Toxicity	0.053	0.019	0.632	0.536
Rat Oral Acute Toxicity	0.748	0.67	0.637	0.388
Carcinogenicity	0.188	0.274	0.108	0.022

**Table 6:** MM-PBSA energies (ΔG_Binding_) for ligands binding at both active sites of EGFR (PDB ID: 3POZ) and HER2 (PDB ID: 3RCD) target receptors. All the energies units in kJ/mol.

System	ΔG_Binding_ (kJ/mol)	Electrostatic (kJ/mol)	Van der Waal (kJ/mol)	Polar Salvation (kJ/mol)	Non-Polar Salvation (kJ/mol)
EGFR-tak-285	−68.33 ± 0.16	−28.05 ± 0.16	−50.93 ± 0.34	29.98 ± 0.27	−11.33 ± 0.19
EGFR-lapatinib	−71.73 ± 0.24	−13.74 ± 0.18	−64.99 ± 0.31	25.59 ± 0.28	−18.59 ± 0.14
EGFR-diosgenin	−67.65 ± 0.13	−23.25 ± 0.33	−54.92 ± 0.23	26.98 ± 0.31	−16.46 ± 0.16
EGFR-monohydroxy spirostanol	−62.73 ± 0.31	−18.74 ± 0.34	−53.99 ± 0.19	24.23 ± 0.30	−14.23 ± 0.14
HER2-tak-285	−72.08 ± 0.23	−11.15 ± 0.26	−69.93 ± 0.32	34.98 ± 0.23	−25.98 ± 0.16
HER2-lapatinib	−80.24 ± 0.21	−7.72 ± 0.22	−78.99 ± 0.15	30.87 ± 0.19	−24.40 ± 0.15
HER2-diosgenin	−78.85 ± 0.33	−10.25 ± 0.16	−72.68 ± 0.24	27.76 ± 0.24	−23.68 ± 0.17
HER2-monohydroxy spirostanol	−69.31 ± 0.19	−10.87 ± 0.18	−65.36 ± 0.34	29.11 ± 0.28	−22.19 ± 0.16

## Data Availability

Data is contained within the article and supplementary material.

## Supplementary Materials

The following supporting information can be downloaded at: https://www.mdpi.com/article/10.3390/ph16050704/s1. Figure S1. (a) Superimposition and (b) 2D Interaction analysis of the co-crystallized ligand TAK-285. (c) Re-docked Ligand (depicted in orange for carbon (C), red for oxygen (O), navy for nitrogen (N), and cyan for fluorine (F)) in the Crystal Structure of the Human EGFR Kinase Domain Complexed with TAK-285 (PDB ID: 3POZ). Figure S2. (a) Superimposition and (b) 2D Interaction analysis of the co-crystallized ligand TAK-285. (c) Re-docked ligand in the crystal structure of the human HER2 kinase domain complexed with tak-285 (PDB ID: 3RCD).
