# Supplementary material for: Diosgenin and Monohydroxy Spirostanol from Prunus amygdalus var amara Seeds as Potential Suppressors of EGFR and HER2 Tyrosine Kinases: A Computational Approach
Source: Pharmaceuticals (Basel). 2023 May 6;16(5):704. doi: 10.3390/ph16050704 (PMC10223344; doi:10.3390/ph16050704)
Supplement: Supplementary file 1 [file pharmaceuticals-16-00704-s001.zip › pharmaceuticals-2291673-supplementary.pdf]

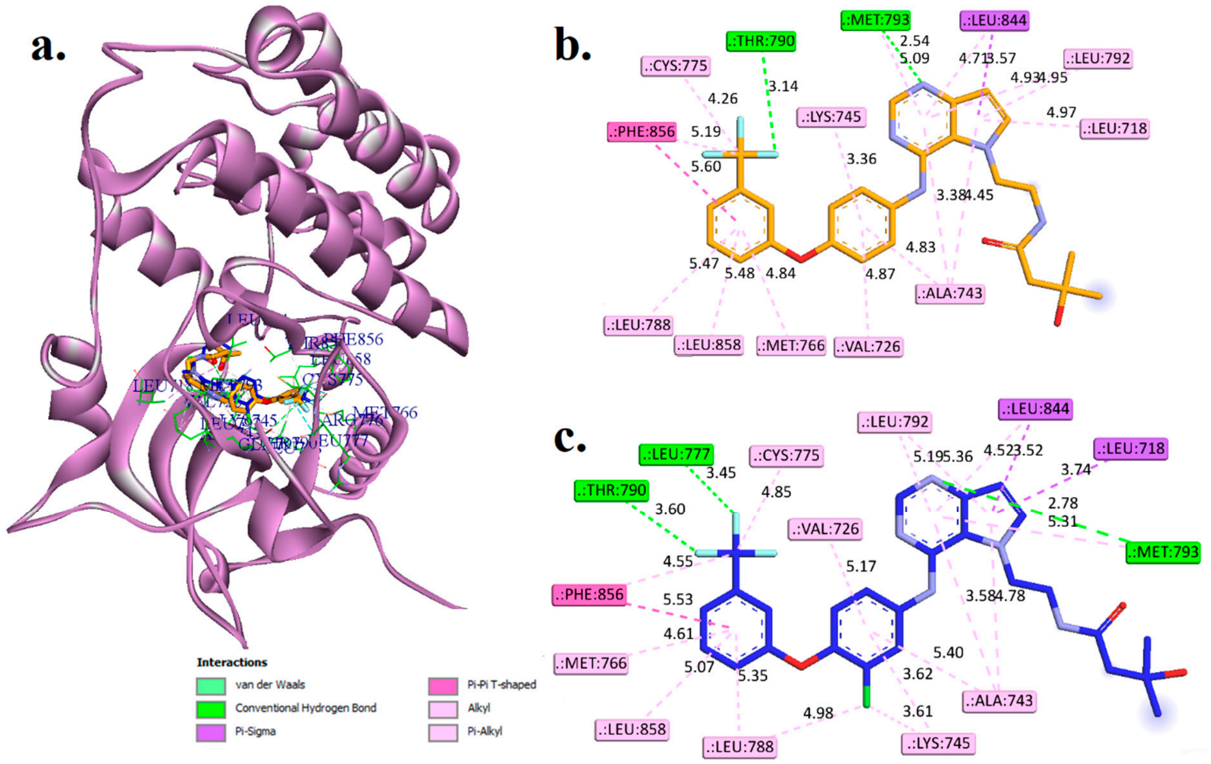

Figure S1. (a) Superimposition and (b) 2D Interaction Analysis of the Co-Crystallized Ligand TAK-285 (depicted in blue for carbon (C), red for oxygen (O), navy for nitrogen (N), and cyan for fluorine (F)). (c) Re-docked Ligand (depicted in orange for carbon (C), red for oxygen (O), navy for nitrogen (N), and cyan for fluorine (F)) in the Crystal Structure of the Human EGFR Kinase Domain Complexed with TAK-285 (PDB ID: 3POZ).

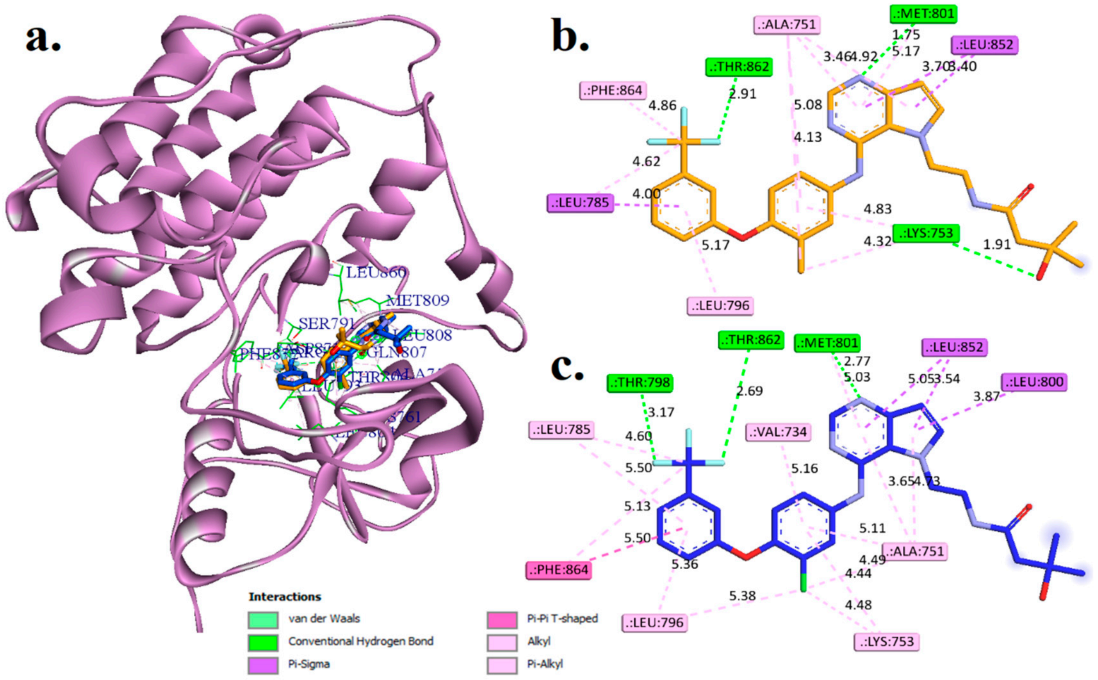

Figure S2. (a) Superimposition and (b) 2D Interaction Analysis of the Co-Crystallized Ligand TAK-285 (depicted in blue for carbon (C), red for oxygen (O), navy for nitrogen (N), and cyan for fluorine (F)). (c) Re-docked Ligand (depicted in orange for carbon (C), red for oxygen (O), navy for nitrogen (N), and cyan for fluorine (F)) in the Crystal Structure of the Human HER2 kinase domain complexed with tak-285 (PDB ID: 3RCD).
